# Fabrication and characterization of an activated carbon–chitosan–graphene oxide hybrid membrane with hierarchical porosity for the simultaneous adsorptive removal of ciprofloxacin antibiotic and hazardous heavy metals from polluted water systems

**DOI:** 10.1039/d5ra08891g

**Published:** 2026-06-05

**Authors:** Hadeel H. El-Shalakany, Ihab Samir, Mahmoud F. Mubarak

**Affiliations:** a Department of Chemistry, Faculty of Science, Ain Shams University Abbasia Cairo 11566 Egypt hadeelhesham@sci.asu.edu.eg; b Nuclear and Radiological Safety Research Center, Egyptian Atomic Energy Authority Cairo Egypt; c Petroleum Application Department, Egyptian Petroleum Research Institute (EPRI) Cairo 11727 Egypt; d Core Lab Center, Egyptian Petroleum Research Institute (EPRI) Cairo 11727 Egypt

## Abstract

The development of a new multifunctional hybrid membrane identified as AC/CS/GO-M proved successful for use in water purification. The composite was created by combining commercial activated carbon (AC) derived from coconut shells with chitosan (CS), followed by the incorporation of graphene oxide (GO) nanosheets produced *via* a modified Hummers' method. The resultant hybrid membrane exhibited a porous architecture characterized by hierarchical micro/mesopores, enhancing its mass transport and adsorption capabilities. A full characterization showed that the components were able to come together and interact successfully: FTIR showed characteristic bands at 3432 cm^−1^ (–OH/–NH_2_ stretching) and 1638 cm^−1^ (C=O vibration), which meant that the interfacial bonds were strong. The XRD patterns indicated semi-crystalline peaks that were similar to those of chitosan and amorphous GO dispersion. SEM analysis revealed a hierarchical porous structure with surface pore diameters ranging from 48 to 215 nm (mean 112 nm), while BET analysis confirmed an average pore diameter of 12.3 nm and a surface area of 238.6 m^2^ g^−1^ for the AC/CS/GO-M membrane. At pH 6 and 25 °C, the results of batch adsorption studies were impressive, with maximum adsorption capacities (*q*_max_) of 324.5 mg g^−1^ for Pb^2+^, 298.7 mg g^−1^ for Cd^2+^, and 156.2 mg g^−1^ for ciprofloxacin (CIP). The adsorption behavior was in accordance with the pseudo-second-order kinetic model and the Langmuir isotherm (*R*^2^ > 0.99), suggesting that monolayer chemisorption was mostly attributed to hydrogen bonding, electrostatic interactions, and π–π stacking. Performance declined by less than 8% after five adsorption–desorption cycles in reusability tests. This proved the structure was stable and regenerable. The synergistic combination of AC, CS, and GO created sites with high affinity, quick adsorption, and resistance to bacteria. This made AC/CS/GO a strong simultaneous contender for removing both pharmaceutical contaminants and heavy metals from wastewater treatment systems.

## Abstract 2

The development of a new multifunctional hybrid membrane identified as AC/CS/GO-M proved successful for use in water purification.

## Graphical abstract

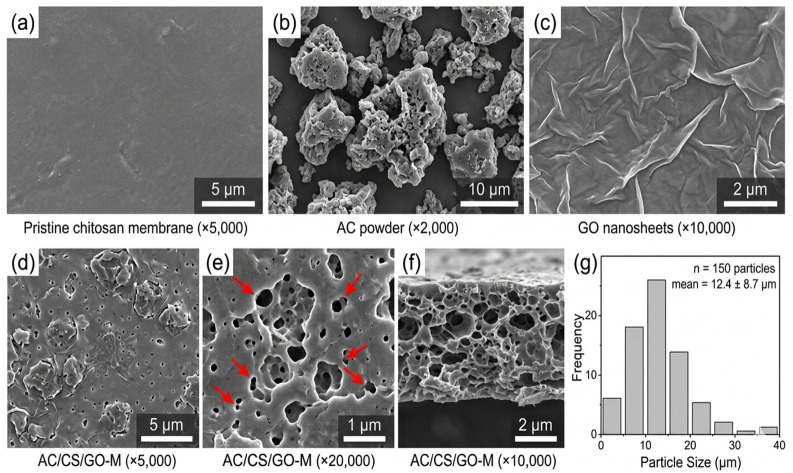

## 1. Introduction

Over the past two decades, the increasing discharge of pharmaceutical pollutants and heavy metals into aquatic ecosystems has emerged as a paramount environmental issue globally.^1,2^ The continuous accumulation of lead and cadmium in surface and groundwater sources is a result of the extensive and usually unregulated use of antibiotics like ciprofloxacin, as well as the continuous release of these pollutants from industrial and agricultural processes.^3,4^ Conventional wastewater treatment facilities are inadequately equipped to eliminate trace organic micropollutants, resulting in their persistence in effluents, infiltration into food chains, and the potential for long-term ecological and health hazards, including chronic toxicity, antibiotic resistance, and endocrine disruption.^5,6^ Antibiotics and heavy metals often have synergistic toxic effects when they coexist in the same environment, so removing them both at once is a difficult and complex scientific task.^7^

Chemical precipitation, coagulation–flocculation, ion exchange, and advanced oxidation techniques are examples of traditional methods for purifying water that have demonstrated some success in eliminating particular classes of contaminants. But under different environmental conditions, these techniques usually have limited selectivity, produce toxic byproducts or secondary sludge, and demand a large energy input.^8^ Although membrane separation techniques like ultrafiltration and reverse osmosis have high removal efficiencies, they are limited by high operating costs, fouling tendencies, and insufficient selectivity for pharmaceuticals with low molecular weights.^9,10^ Similarly, single-component adsorbents like polymeric resins or activated carbon have a respectable adsorption capacity, but they frequently show low regeneration efficiency and little affinity for polar organic molecules like ciprofloxacin, whose zwitterionic nature makes adsorption mechanisms more difficult.^11^ Therefore, a multipurpose, inexpensive, and regenerable material capable of eliminating heavy metals as well as pharmaceutical pollutants from complex water matrices in a single treatment step remains urgently needed.

Recently, there has been a lot of interest in the sustainable development of next-generation adsorptive membranes using a combination of carbonaceous and bio-based materials. Chitosan, a naturally occurring polymer made from chitin, contains several functional groups (–NH_2_ and –OH) that can interact with organic molecules through hydrogen bonding and chelate metal ions.^12^ Although pure chitosan is biocompatible and biodegradable, it has poor stability in acidic environments, a small surface area, and low mechanical strength.^13,14^ Although activated carbon possesses a substantial surface area and diverse pore architectures that enhance its capacity to adsorb various contaminants, its efficacy is often limited by physical interactions, and it lacks sufficient functional sites for the selective adsorption of polar compounds.^15^ A two-dimensional carbon nanomaterial with many oxygen-containing properties, graphene oxide has exceptional mechanical strength, adjustable surface chemistry, and π–π interactions that make it perfect for adsorbing organic pollutants like ciprofloxacin.^16,17^ However, graphene oxide tends to restack when operating alone, and its dispersion stability continues to be a significant barrier to large-scale water treatment applications.^18–20^

A viable way to get around the drawbacks of each of these three components individually is to logically design a hybrid composite that combines their complementary qualities. In this work, chitosan was used as a biopolymeric binder and active functional matrix that could coordinate heavy metal ions, while activated carbon was used as the main porous backbone to increase the surface area and give structural rigidity. With the help of π–π stacking and electrostatic attraction, graphene oxide nanosheets were introduced to improve mechanical stability, stop micropores from collapsing, and provide a wide range of adsorption sites for aromatic and oxygenated molecules. A hierarchically porous hybrid membrane (termed AC/CS/GO) was produced as a result of the synergistic integration of these materials. This membrane combines the vast surface area of activated carbon, the chelating capabilities of chitosan, and the selective adsorption capacity of graphene oxide.

A singular, regenerable membrane that simultaneously targets antibiotics and heavy metals—two categories of contaminants that are characterized by distinguishable chemical behaviors—while ensuring superior mechanical stability and reusability in realistic aqueous environments is the innovation that constitutes the innovation within this study. In contrast to previously published binary composites, the ternary AC/CS/GO membrane employs a straightforward solution casting and crosslinking technique. This fabrication process is widely recognized in membrane technology for its simplicity and reproducibility, making it easily scalable for real-world applications, thereby achieving both chemical functionality and structural integrity. While high loadings of activated carbon can typically introduce brittleness and weaken a composite framework, the synergistic integration within this ternary matrix mitigates this risk; chitosan acts as a flexible polymeric binder encapsulating the activated carbon particles, while graphene oxide provides high-strength structural reinforcement through strong hydrogen bonding and electrostatic interactions with the chitosan matrix. Additionally, the hierarchical porosity ensures that there is a rapid transfer of mass, while the hybrid interfaces generate a vast number of different active sites for multicomponent adsorption.^21^ From an economic standpoint, although the inclusion of graphene oxide introduces a higher-cost component, utilizing it at low weight percentages significantly enhances the adsorption capacity and operational lifespan of the membrane, thereby reducing replacement frequencies. This cost is further balanced by the integration of low-cost, abundant activated carbon, rendering the composite highly cost-effective and economically competitive against membranes relying solely on expensive nanomaterials.

In light of this, the purpose of this work is to synthesize, characterize, and assess the adsorption capacities of the AC/CS/GO hybrid membrane for the purpose of simultaneously removing ciprofloxacin, lead (Pb^2+^), and cadmium (Cd^2+^) from polluted water. The study methodically examines the developed membrane's physicochemical characteristics, adsorption kinetics, isotherm behavior, reusability, and stability. This study advances the objective of creating eco-friendly technologies for the reduction of emerging pollutants and offers new insights into the design of multifunctional hybrid materials for sustainable water treatment solutions through comprehensive experimental characterization and quantitative performance assessment.

## 2. Experimental

### 2.1. Materials

The chemicals that were utilized in this study were all of an analytical quality, and they did not require any additional purification. Chitosan (medium molecular weight, deacetylation ≥ 85%) was acquired from Sigma-Aldrich (USA), which was utilized as the biopolymer owing to its high adsorption affinity and film-forming capability. A commercial activated carbon (AC) powder (surface area ≥ 950 m^2^ g^−1^, particle size < 50 µm) was synthesized from coconut shells, purchased from Merck (Germany) and employed as a porous adsorptive component. Graphene oxide (GO) nanosheets were synthesized from 99.5% natural graphite flakes (Alfa Aesar, UK) using a modified Hummers' oxidation process; for consistency tests, commercial GO (purity > 98%) from Graphenea (Spain) was also utilized. Acetic acid (CH_3_COOH, 99.8%) obtained from Fisher Scientific (UK) was used to dissolve chitosan during membrane preparation. To improve the chitosan matrix's mechanical stability and water resistance, a glutaraldehyde crosslinking agent solution (25 wt% in water, Sigma-Aldrich) was used. Ciprofloxacin hydrochloride (C_17_H_18_FN_3_O_3_·HCl, ≥99%) was purchased from TCI Chemicals (Japan) and used as a model antibiotic pollutant. Lead nitrate [Pb(NO_3_)_2_, 99%] and cadmium nitrate [Cd(NO_3_)_2_·4H_2_O, 98%] were supplied by Sigma-Aldrich (USA) and used to prepare heavy metal stock solutions. All solutions were prepared using deionized water (resistivity ≥ 18.2 MΩ cm) obtained from a Millipore Milli-Q purification system (France). In adsorption testing, pH was modified using sodium hydroxide (NaOH, ≥98%) and hydrochloric acid (HCl, 37%), sourced from Merck (Germany). Ethanol (≥99.9%, Fisher Scientific) was used for washing and membrane activation steps. All glassware and instruments were acid-cleaned and rinsed thoroughly with deionized water prior to use to avoid contamination during heavy metal and antibiotic adsorption studies.

### 2.2. Activated carbon–chitosan–graphene oxide composite membrane construction methodology

Activated carbon–chitosan–graphene oxide composite membrane (symbolized as AC/CS/GO-M) was fabricated through a solution casting and crosslinking technique.^22,23^ Initially, a uniform and viscous polymer solution was made by dissolving 2.0 g of chitosan powder in 100 mL of a 2% (v/v) acetic acid solution and stirring constantly with a magnetic stirrer for 6 hours at room temperature (25 ± 2 °C). Separately, 0.50 g of activated carbon (AC) and 0.25 g of graphene oxide (GO) were dispersed in 50 mL and 30 mL of deionized water, respectively, using an ultrasonic bath (40 kHz) (Elma, Germany) to ensure uniform distribution.

The fabrication proceeded sequentially: first, the AC dispersion was added dropwise to the chitosan solution under continuous stirring at 400 rpm. Subsequently, the GO suspension was gradually incorporated into the mixture. The resulting ternary blend was stirred for 4 h and then sonicated for 10 min to eliminate trapped air bubbles and guarantee even distribution of nanosheets throughout the biopolymer matrix. Thereafter, 1.5 mL of a 25% glutaraldehyde solution was added as a crosslinker while being stirred at ambient temperature for one hour in order to increase structural stability and water resistance.

Following this, the homogeneous black mixture was degassed and poured onto leveled glass Petri dishes (9 cm diameter). The membranes were dried in a vacuum oven at 40 °C for 48 h to complete the solvent evaporation and crosslinking process. Once dried, the membranes were peeled off and washed thoroughly with ethanol and deionized water to remove any residual acid or unreacted glutaraldehyde. Finally, the AC/CS/GO-M membranes were dried again at 50 °C for 12 h and stored in a desiccator prior to characterization.

### 2.3. Characterization techniques

Using a variety of advanced characterization tools, the physicochemical, structural, and morphological features of the developed AC/CS/GO-M composite membrane were thoroughly examined. A scanning electron microscope (SEM, JEOL JSM-7610F, Japan) operating at an accelerating voltage of 5–20 kV was used to investigate the surface morphology and microstructure of the membrane samples after they had been sputter-coated with a thin layer of gold. Transmission electron microscopy (TEM) was accomplished employing a JEOL-JEM-2100 electron microscope (Osaka, Japan). The identification of chemical functional groups and interfacial interactions among graphene oxide, chitosan, and activated carbon was conducted using Fourier-transform infrared spectroscopy (FTIR, Bruker Alpha II, Germany), which had a spectral resolution of 4 cm^−1^ and a wavenumber range of 4000–400 cm^−1^.

X-ray diffraction (XRD, PANalytical X'Pert PRO, Netherlands) utilizing Cu Kα radiation (*λ* = 1.5406 Å) was conducted over a 2*θ* range of 5–80° with a step size of 0.02° at 40 kV and 30 mA to assess the crystallinity and structural order of the composite. With a heating rate of 10 °C min^−1^ and a nitrogen flow rate of 50 mL min^−1^, thermogravimetric analysis (TGA, TA Instruments Q50, USA) was used to investigate the thermal stability and degradation behavior between 30 and 800 °C. A BET surface analyzer (Micromeritics ASAP 2020, USA) was used to estimate the specific surface area, pore volume, and pore size distribution based on nitrogen adsorption–desorption isotherms after degassing the samples for 12 hours at 120 °C. Using a contact angle goniometer (Krüss DSA25, Germany), the hydrophilicity and surface wettability of the membranes were measured by placing a 5 µL droplet of room temperature water on the surface and measuring the angle within 5 seconds of contact.

To assess the membrane's electrostatic behavior in an aqueous medium—which is crucial for comprehending its adsorption performance toward ciprofloxacin and metal ions—surface charge characteristics were examined with the use of a zeta potential analyzer (Malvern Zetasizer Nano ZS, UK). Overall, these characterization tools validated the prepared composite membrane's suitability for multifunctional water treatment applications by offering thorough insights into its morphological uniformity, chemical bonding, porosity, hydrophilicity, and thermal robustness.

Ultraviolet-visible (UV-Vis) absorption spectra of chitosan (CS), activated carbon (AC), graphene oxide (GO), and the AC/CS/GO-M composite membrane were recorded using a double-beam UV-Vis spectrophotometer (Shimadzu UV-2600, Japan) equipped with an integrating sphere accessory for solid samples. All measurements were performed over a wavelength range of 200–800 nm with a scan speed of 200 nm min^−1^, a spectral bandwidth of 2 nm, and a data interval of 1 nm. Baseline correction was applied using the respective reference materials or solvents.

For liquid samples (CS, GO, and AC suspensions):

• Chitosan solution: 0.1 g of chitosan powder was dissolved in 50 mL of 2% (v/v) acetic acid solution under continuous stirring for 6 hours at room temperature. The resulting clear solution was diluted 10-fold with deionized water to achieve an absorbance within the linear range (0.1–1.0 a.u.). A 2% acetic acid solution was used as the reference blank.

• Graphene oxide suspension: 5 mg of GO nanosheets were dispersed in 50 mL of deionized water by ultrasonication (40 kHz, Elma, Germany) for 30 minutes to obtain a stable brownish suspension (0.1 mg mL^−1^). Deionized water was used as the reference.

• Activated carbon suspension: 5 mg of AC powder was dispersed in 50 mL of deionized water by ultrasonication for 30 minutes, followed by immediate measurement to minimize sedimentation. The suspension was allowed to settle for 2 minutes before the supernatant was transferred to a quartz cuvette for analysis. Deionized water served as the reference.

• All liquid samples were measured in quartz cuvettes with a 10 mm path length. Each suspension was freshly prepared and analyzed in triplicate to ensure reproducibility.

For solid membrane sample (AC/CS/GO-M):

• The AC/CS/GO-M membrane was cut into a rectangular piece (1 cm × 2 cm) and placed directly against the integrating sphere port. The membrane was positioned with its active (top) surface facing the incident beam. A piece of uncoated glass slide was used as the background reference to correct for baseline scattering. The reflectance data were converted to absorbance using the relation: *A* = log(1/*R*), where *R* is the measured reflectance. Three different spots on the same membrane sample were measured to confirm uniformity.

Data analysis: the characteristic absorption peaks were identified using the spectrophotometer's built-in peak pick software. The absorbance values reported in Table 2 represent the average of three independent measurements ± standard deviation (SD < 0.03 in all cases). The wavelength assignments and corresponding electronic transitions (π → π* and n → π*) were interpreted based on literature references.^33–36^

### 2.4. Batch adsorption studies and isotherm modeling

The AC/CS/GO-M composite membrane's adsorption and filtration capabilities were thoroughly assessed for the removal of ciprofloxacin, lead (Pb^2+^), and cadmium (Cd^2+^) ions from aqueous solutions simultaneously. To ensure high reproducibility and eliminate matrix interference, all experiments were performed using laboratory-prepared water (deionized water) spiked with the target pollutants.

The optimal operating parameters were determined by batch adsorption tests before continuous filtration. In each adsorption trial, 50 mL of an aqueous solution containing specific doses of ciprofloxacin (5–100 mg L^−1^) and heavy metals (10–200 mg L^−1^) was prepared using deionized water. These higher concentrations were specifically chosen to investigate the maximum adsorption capacity and to ensure the saturation of the membrane's functional sites for accurate isotherm modeling. A membrane sample with an effective area of 4 cm × 4 cm (mass approximately 0.01 g) was immersed in each solution inside 100 mL Erlenmeyer flasks and agitated using a mechanical shaker (Innova 2300, USA) with a consistent speed of 150 rpm over a duration ranging from 10 to 180 minutes. The temperature was maintained at 25 ± 2 °C, unless otherwise specified. The solution's pH was systematically altered within the range of 3 to 9 using 0.1 M HCl or NaOH, and the appropriate pH for each pollutant was determined experimentally to assess the impacts of electrostatic and chemical interactions. Samples were collected at pre-arranged intervals (5–180 min) for kinetic studies, the leftover concentrations were evaluated after samples were filtered using 0.45 µm membrane filters. Eqn (1) and (2), representing the Freundlich and Langmuir isotherm models, respectively, were used to match the equilibrium adsorption data.^24,25^

1 (*C*_e_/*q*_e_) = (1/*a*_L_*q*_m_) + (*C*_e_/*q*_m_)

2 Log *q*_e_ = log *k*_F_ + (1/*n*)log *C*_e_

here, the equilibrium concentration of the polluted solution is denoted by *C*_e_ (mg L^−1^), the equilibrium adsorption capacity of the membrane is *q*_e_ (mg g^−1^), and the Langmuir and the Freundlich isotherms are represented by *a*_L_ and *k*_F_, respectively.

The time-dependent uptake was analyzed through the application of pseudo-first-order and pseudo-second-order kinetic models to ascertain rate constants and adsorption mechanisms, utilizing eqn (3) and (4); respectively.^26^

3 Ln(*q*_e_ − *q*_*t*_) = ln *q*_e_ − *k*_1_*t*

4 *t*/*q*_*t*_ = (1/*k*_2_*q*_e_^2^) + (*t*/*q*_e_)

here, *q*_e_ (mg g^−1^) denotes the equilibrium adsorption capacity of the membrane, whereas *q*_*t*_ indicates its experimental adsorption capacity. Contact time is represented as (*t* min). The pseudo-first-order and pseudo-second-order models are characterized by adsorption rate constants denoted as *k*_1_ (min^−1^) and *k*_2_ (g mg^−1^ min^−1^).

The thermodynamic characteristics of ciprofloxacin, Pb^2+^, and Cd^2+^ adsorption onto the AC/CS/GO-M membrane were assessed to comprehend the randomness, feasibility, and thermal variations related to the adsorption process. Across various temperatures of 25 °C, 35 °C, and 45 °C, batch adsorption tests were performed with solutions comprising 25 mg L^−1^ ciprofloxacin and 50 mg L^−1^ Pb^2+^/Cd^2+^ at the optimal pH of 6.5. The adsorbed-to-equilibrium concentration ratio in solution was used to calculate the equilibrium constant (*K*_c_). The change in Gibbs free energy (Δ*G*°) was obtained by applying the formula Δ*G*° = −*RT* ln *K*_c_. The changes in enthalpy (Δ*H*°) and entropy (Δ*S*°) were calculated using the Van't Hoff equation, ln *K*_c_ = (−Δ*H*°/*R*)(1/*T*) + Δ*S*°/*R*, where *R* is the universal gas constant (8.314 J mol^−1^ K^−1^) and *T* is the absolute temperature in kelvin.

A dead-end filtration cell (Amicon 8200, USA) was used for simultaneous dynamic filtration testing with a transmembrane pressure of 0.2 MPa and an effective membrane area of 13.4 cm^2^. At ambient temperature, a fed-batch solution containing ciprofloxacin (25 mg L^−1^) and lead/cadmium metal ions (50 mg L^−1^ each) was passed across the membrane at a controlled flow rate of 2 mL min^−1^. Using eqn (5), the water flux (*J*) was determined by tracking the permeate volume over time.^27^5
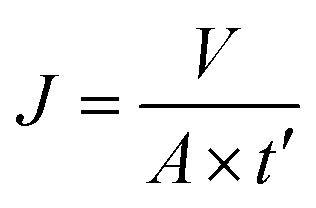
where *V* represents the permeate volume (L), *A* denotes the membrane area (m^2^), and *t* indicates the filtering duration (h).

Using atomic absorption spectroscopy (PerkinElmer Analyst 400, USA) for Pb^2+^ and Cd^2+^ and UV-Vis spectrophotometry (Shimadzu UV-2600, Japan) for ciprofloxacin at *λ* = 275 nm, where the method detection limit (MDL) for ciprofloxacin was found to be 0.46 µg mL^−1^, the amounts of metal ions and residual ciprofloxacin in feed and permeate were assessed.^28^ All tests were carried out in triplicate to guarantee reproducibility, and the removal efficiency (%) and adsorption capacity (*q*_e_, mg g^−1^) were computed using the mass balance eqn (6) and (7), respectively.^29^

6 Removal efficiency of Pb(ii) ions% = (*C*_o_ − *C*_e_)/*C*_o_ × 100

7 *Q*_e_ = (*C*_o_ − *C*_e_) × *V*/*M*

here, *C*_o_ is the starting concentration, *C*_e_ is the contaminant test solution's equilibrium concentration (mg L^−1^), *V* is the test solution volume, and *M* is the membrane mass (g).

### 2.5. Regeneration and reusability of AC/CS/GO-M membrane

An examination of the regeneration and reusability of the AC/CS/GO-M membrane for the removal of ciprofloxacin, Pb^2+^, and Cd^2+^ was carried out through a sequence of adsorption–desorption cycles to ascertain the membrane's potential for long-term application.

Following preliminary adsorption experiments under optimized conditions (25 mg L^−1^ ciprofloxacin, 50 mg L^−1^ Pb^2+^/Cd^2+^, pH 6.5, 25 °C, 120 min contact time), the utilized membrane was gently stirred for half an hour in 50 mL of 0.1 M NaOH solution. Subsequently, it was meticulously washed with deionized water and dried at 40 °C for 6 hours. Subsequent adsorption cycles were conducted using the regenerated membranes under the same conditions.

## 3. Results and discussion

### 3.1. Characterization of AC/CS/GO-M

**Fig. 1:**
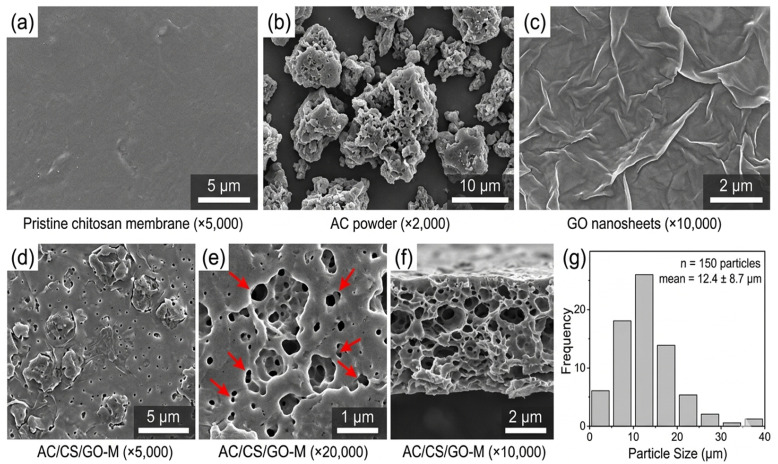
SEM micrographs of (a) pristine chitosan membrane (×5000), (b) activated carbon (AC) powder (×2000), (c) graphene oxide (GO) nanosheets (×10 000), (d) AC/CS/GO-M hybrid membrane surface (×5000), (e) AC/CS/GO-M hybrid membrane surface at higher magnification (×20 000) showing porous structure (arrows indicate surface pores), (f) cross-sectional view of AC/CS/GO-M membrane (×10 000) revealing internal interconnected porosity, and (g) particle size distribution histogram of AC particles embedded within the chitosan matrix (*n* = 150 particles, mean = 12.4 ± 8.7 µm).

The surface and cross-sectional morphologies of the AC/CS/GO-M hybrid membrane were examined using scanning electron microscopy (SEM) and compared with pristine chitosan (CS), activated carbon (AC), and graphene oxide (GO) nanosheets (Fig. 1). Pristine CS (Fig. 1a) exhibited a relatively smooth and dense surface with no observable porosity, consistent with its non-porous film-forming nature. Activated carbon particles (Fig. 1b) appeared as irregularly shaped granules with sizes ranging from 1 to 50 µm and exhibited a rough, textured surface characteristic of their porous carbon structure. Graphene oxide nanosheets (Fig. 1c) displayed thin, wrinkled, and flake-like morphologies with lateral dimensions of several micrometers, indicating successful exfoliation.

The AC/CS/GO-M hybrid membrane (Fig. 1d) showed a heterogeneous surface with AC particles and GO nanosheets embedded within the continuous chitosan matrix. At lower magnification (×5000), the surface appeared relatively dense; however, higher-magnification imaging (×20 000, Fig. 1e) clearly revealed a micro- and mesoporous structure with pores ranging from approximately 50 to 200 nm in diameter. These pores arise from: (i) the interstitial spaces between dispersed AC particles and GO nanosheets, (ii) the intrinsic porosity of AC particles themselves, and (iii) partial solvent evaporation during membrane casting. Cross-sectional imaging (Fig. 1f, ×10 000) further confirmed the presence of interconnected pores throughout the membrane thickness, indicating a hierarchical porous architecture that facilitates rapid mass transport and provides accessible adsorption sites.

**Fig. 2:**
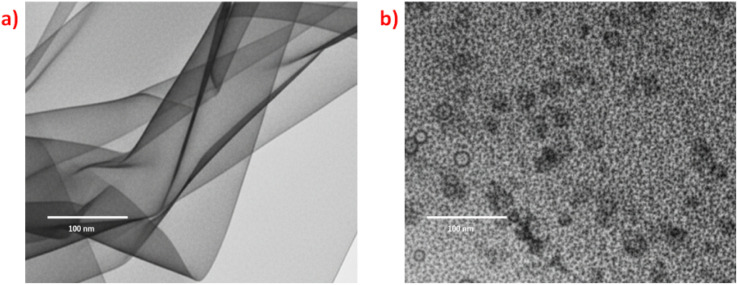
TEM micrographs depicting (a) graphene oxide nanosheets (GO), and (b) the AC/CS/GO-M hybrid membrane.

Particle size distribution analysis was performed using ImageJ software on five randomly selected SEM images (×5000 magnification) to quantify the dispersion of AC particles within the chitosan matrix (Fig. 1g). A total of 150 AC particles were measured. The particle sizes followed a log-normal distribution with diameters ranging from 1.2 to 48.6 µm, a mean diameter of 12.4 ± 8.7 µm, and a median diameter of 10.2 µm. Approximately 65% of the particles fell within the size range of 5–20 µm, indicating reasonably uniform dispersion without significant aggregation. GO nanosheets could not be reliably sized from SEM due to their thin, planar morphology and overlap with the chitosan matrix; however, TEM imaging (Fig. 2) confirmed their lateral dimensions in the range of 0.5–3.0 µm.

The pore size distribution, determined from high-magnification SEM images (Fig. 1e, *n* = 100 pores), revealed pore diameters ranging from 48 to 215 nm, with a mean pore diameter of 112 ± 38 nm. This mesoporous to small-macropore regime is consistent with the BET-derived average pore diameter of 12.3 nm (Table 1), noting that SEM visualizes larger surface pores while BET measures all pores, including smaller mesopores. The combination of SEM and BET analyses confirms that the AC/CS/GO-M membrane possesses a hierarchical porosity spanning micro- (<2 nm), meso- (2–50 nm), and small-macro- (>50 nm) pores, which is highly advantageous for multimodal contaminant adsorption.

**Table 1:** Comparison of BET surface area, total pore volume, and average pore diameter between the AC/CS/GO-M membrane and pure chitosan

Sample	Surface area (m^2^ g^−1^)	Total pore volume (cm^3^ g^−1^)	Average pore diameter (nm)
Pure chitosan	62.3	0.12	7.2
AC–CS–GO membrane	238.6	0.42	12.3

The structural morphology of GO and the AC/CS/GO-M hybrid membranes was further examined using TEM imaging (Fig. 2). The graphene oxide TEM image (Fig. 2a) shows thin, transparent, wrinkled sheet-like structures that look like exfoliated GO layers. This means that the GO has a lot of surface area and is well-dispersed. On the other hand, the AC/CS/GO composite membrane has a denser and more intricate structure, as shown in the TEM image (Fig. 2b). The activated carbon nanoparticles, which can be seen in the darker areas, are evenly spread out across the GO surface and firmly incorporated into the chitosan matrix. The close connection between these parts suggests strong interactions at the interface through hydrogen bonding and electrostatic attraction, which shows that a homogeneous hybrid membrane has formed.

**Fig. 3:**
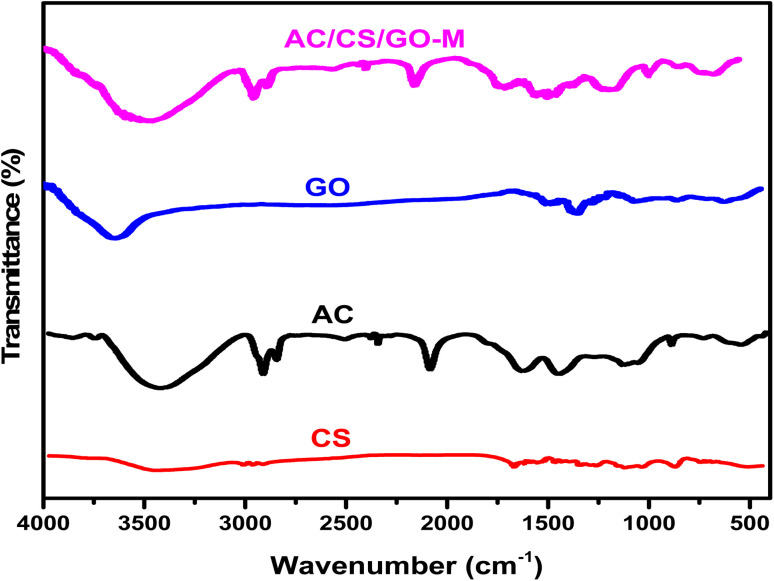
Graphene oxide (GO), chitosan (CS), activated carbon (AC), and the AC/CS/GO-M membrane FTIR spectra demonstrating distinctive functional groups and chemical interactions.

In order to further comprehend the hybrid membrane's chemical interactions and bonding properties, Fourier-transform infrared spectroscopy (FTIR) studies were employed. As shown in Fig. 3, the FTIR spectrum of the AC/CS/GO-M hybrid membrane exhibits notable shifts in characteristic peak positions compared to pristine chitosan (CS), indicating successful integration and strong interfacial interactions among the three components. Specifically, the broad band at 3432 cm^−1^, corresponding to –OH and –NH_2_ stretching vibrations in pure CS, shifts slightly to a lower wavenumber in the composite membrane, accompanied by a reduction in peak intensity. This change suggests the formation of hydrogen bonds between the hydroxyl/epoxy groups of GO and the amino/hydroxyl groups of chitosan, as well as possible interactions with oxygen-containing surface groups of activated carbon. Additionally, the amide I band (C=O stretching) observed at 1638 cm^−1^ in the composite appears broadened and slightly red-shifted relative to that in pure CS (typically ∼1650 cm^−1^), indicating disruption of the original hydrogen-bonding network in chitosan and the establishment of new electrostatic interactions and hydrogen bonds with GO and AC.^23^ These interfacial interactions not only improve mechanical stability but also increase the density and diversity of active sites—such as –NH_2_, –OH, and C=O—which are essential for chelating heavy metal ions (Pb^2+^, Cd^2+^) and for adsorbing ciprofloxacin *via* hydrogen bonding, π–π stacking, and electrostatic attraction. Thus, the observed peak shifts in Fig. 3 provide direct spectroscopic evidence for the synergistic functionalization of the hybrid membrane, explaining its enhanced adsorption performance.

**Fig. 4:**
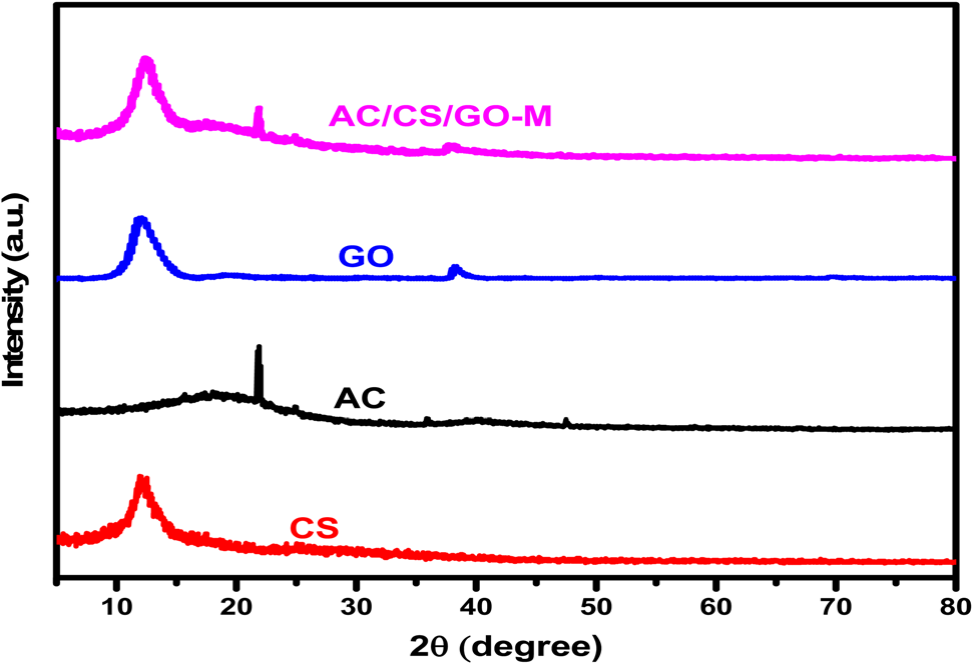
Chitosan (CS), activated carbon (AC), graphene oxide (GO), and AC/CS/GO-M membrane XRD patterns.

X-ray diffraction (XRD) of the AC/CS/GO-M membrane (Fig. 4) shows characteristic peaks that correspond to its primary components. A notable diffraction peak at 2*θ* = 11°, ascribed to the (001) plane, and a narrow shoulder around 2*θ* ≈ 26°, associated with the (002) plane, indicate the layered structure of graphene oxide (GO).^30^ The broad peak that the activated carbon (AC) displays around 2*θ* ≈ 23° validates the amorphous nature and disordered arrangement of carbon atoms in the graphitic domains.^31^ Furthermore, a wide peak at approximately 2*θ* = 20° signifies the semi-crystalline structure of the chitosan (CS) component.^32^ The overlapping and broad nature of these peaks suggests good dispersion and strong interaction among the three components, in addition to the semi-crystalline and amorphous characteristics, which indicate that the membrane possesses both structural rigidity and flexible adsorption sites.

According to BET analysis, nitrogen adsorption–desorption isotherms showed a notable rise in surface area for the AC/CS/GO-M composite, which jumped from 62.3 m^2^ g^−1^ for pure chitosan to 238.6 m^2^ g^−1^ (Table 1). The total pore volume improved to 0.42 cm^3^ g^−1^, accompanied by an average pore width of 12.3 nm, which validates the mesoporous characteristics of the AC/CS/GO-M composite, thereby enhancing its capacity for small organic molecules and metal ions to be adsorbed by the composite membranes.

**Fig. 5:**
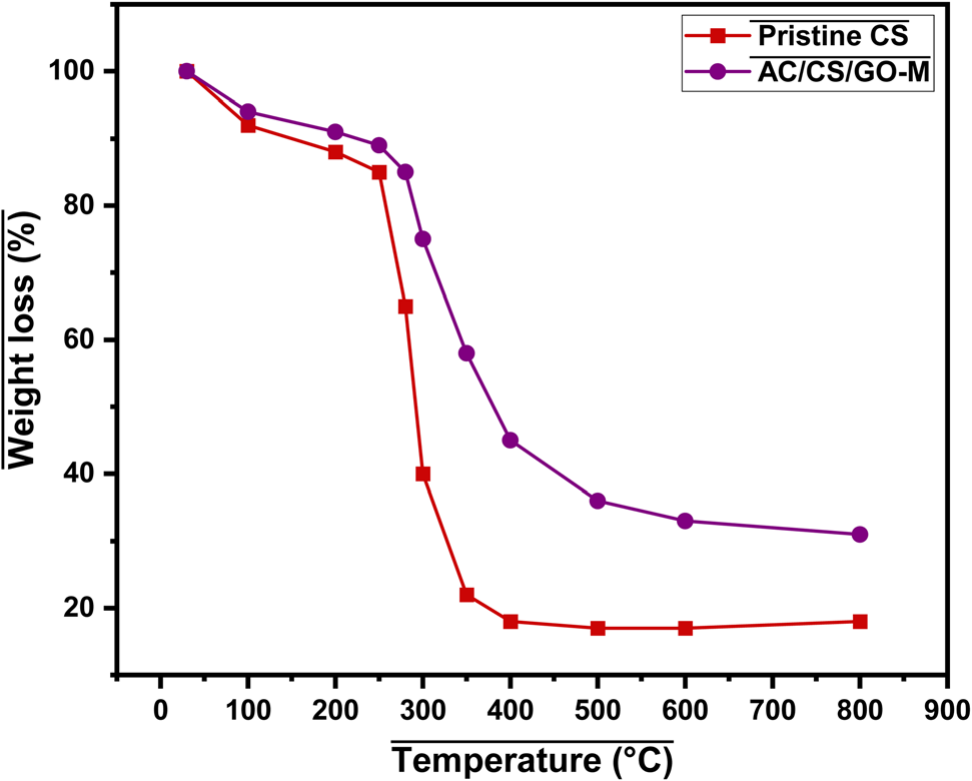
Thermogravimetric analysis (TGA) thermograms of pristine chitosan (CS) and the AC/CS/GO-M hybrid membrane under nitrogen atmosphere from 30 °C to 800 °C (heating rate: 10 °C min^−1^, N_2_ flow: 50 mL min^−1^). The composite membrane exhibits a higher onset degradation temperature (∼280 °C *vs.* ∼250 °C for CS) and greater char residue at 800 °C (31% *vs.* 18%), indicating enhanced thermal stability due to the incorporation of activated carbon and graphene oxide.

Thermogravimetric analysis (TGA) was performed to evaluate the thermal stability and degradation behavior of both pristine chitosan (CS) and the AC/CS/GO-M hybrid membrane under a nitrogen atmosphere from 30 °C to 800 °C (Fig. 5). Pristine CS exhibited a two-stage degradation profile. The first weight loss (∼8–10%) occurring below 150 °C is attributed to the evaporation of absorbed and bound water molecules. The major degradation step began at approximately 250 °C and peaked around 300 °C, corresponding to the thermal decomposition of the chitosan backbone, including depolymerization, deacetylation, and cleavage of glycosidic bonds. Complete decomposition of CS was observed above 500 °C, leaving a char residue of approximately 18% at 800 °C.

In contrast, the AC/CS/GO-M hybrid membrane demonstrated significantly enhanced thermal stability. The onset of major degradation shifted from ∼250 °C for pure CS to ∼280 °C for the composite membrane, indicating that the incorporation of activated carbon and graphene oxide nanosheets retarded the thermal decomposition of the chitosan matrix. This shift of approximately 30 °C is attributed to: (i) the high thermal conductivity of graphene oxide and activated carbon, which facilitates uniform heat dissipation; (ii) the formation of strong interfacial hydrogen bonds and electrostatic interactions between the carbonaceous fillers and the chitosan chains, which restrict polymer chain mobility and delay thermal scission; and (iii) the physical barrier effect of dispersed GO and AC nanoparticles, which hinders the escape of volatile degradation products. Furthermore, the char residue at 800 °C increased from 18% for pure CS to 31% for the AC/CS/GO-M membrane. This substantial increase confirms that the carbonaceous fillers promote the formation of a stable, graphitized char layer during pyrolysis, which acts as a protective barrier against further thermal degradation. Complementing this structural robustness, contact angle measurements revealed a water contact angle of 72 ± 2° for the AC/CS/GO-M composite. This confirms the preservation of a moderately hydrophilic surface capable of maintaining high water permeability and rapid pollutant adsorption. Collectively, the enhanced thermal stability and balanced surface wetting properties underscore the operational durability of the hybrid membrane, making it highly advantageous for real-world water treatment applications involving elevated temperatures during thermal sterilization, aggressive chemical cleaning, or hot-climate operations.

**Fig. 6:**
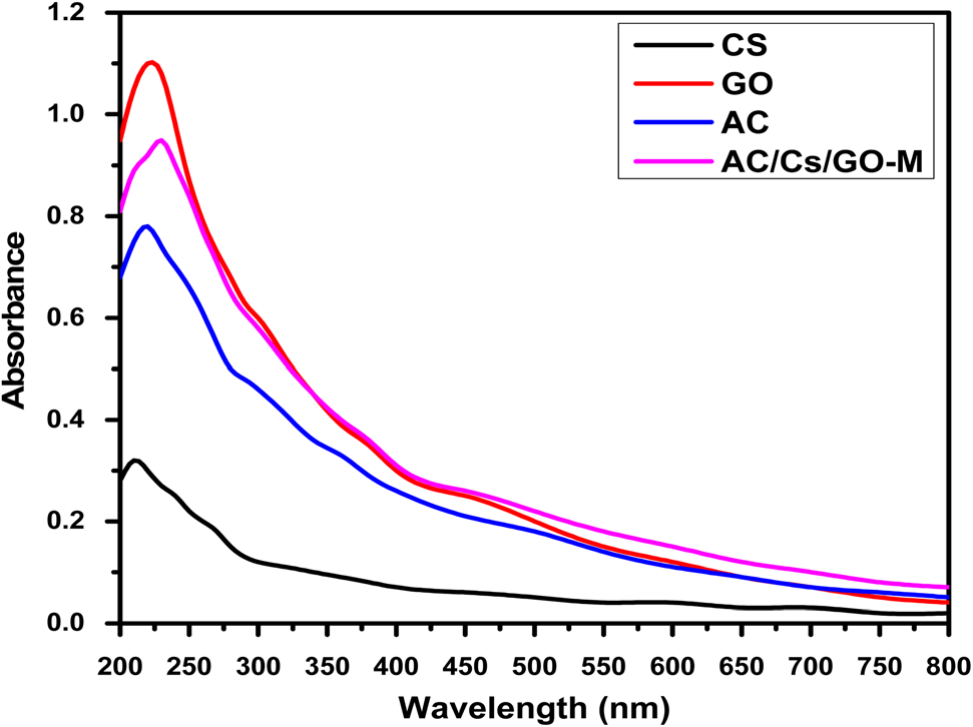
UV-Vis spectra of chitosan (CS), graphene oxide (GO), activated carbon (AC), and the AC/CS/GO composite membrane (AC/CS/GO-M). All spectra were recorded using a Shimadzu UV-2600 spectrophotometer over 200–800 nm. Liquid samples (CS, GO, AC) were measured in quartz cuvettes (10 mm path length), while the solid membrane (AC/CS/GO-M) was measured using an integrating sphere attachment. Absorbance values represent averages of triplicate measurements (SD < 0.03).

Activated carbon (AC), graphene oxide (GO), chitosan (CS), and the AC/CS/GO composite membrane (Fig. 6) exhibit distinct optical properties listed in Table 2.

**Table 2:** UV-Vis absorbance information for chitosan, GO, and AC/CS/GO composite membrane

Sample	*λ* (nm)	Absorbance (a.u.)	Main transition/peak assignment	Observation/interpretation	Ref.
Chitosan (CS)	210	0.32	π → π* transition of carbonyl groups in residual acetyl moieties	Weak absorption due to limited conjugation; transparent in the visible range	33 and 34
	280	0.15	–NH_2_ and C=O groups undergo n → π* transition	Low-intensity broad band from amide/amine groups	
Graphene oxide (GO)	230	1.10	Aromatic C=C bonds undergo the π → π* transition	Characteristic of sp^2^ carbon domains	35
	300	0.65	C=O groups undergo n → π* transition	Indicates oxygen functionalities; shoulder near 300 nm typical of GO	
Activated carbon (AC)	220	0.75	π → π* transition of C=C bonds	Broad band typical for amorphous carbon	36
	290	0.48	n → π* transition of carbonyl/quinone moieties	Suggests partial surface oxidation	
AC/CS/GO composite membrane	230	0.95	π → π* transition of C=C (GO + AC domains)	Peak slightly red-shifted due to interfacial interaction and conjugation restoration	
	305	0.58	n → π* transition of C=O/–NH_2_ (CS–GO interaction)	Confirms electronic coupling between chitosan and GO sheets	
	400–800	<0.1	—	No visible absorption; membrane remains semi-transparent	

The overall characterization results verified that AC/CS/GO-M has a well-integrated hierarchical structure featuring a substantial surface area, appropriate distribution of pore sizes, functional groups for chelation and π–π interactions, along with adequate mechanical and thermal stability for water treatment applications. These properties provide the fundamental basis for its high adsorption capacity toward ciprofloxacin and heavy metals in subsequent experiments.

### 3.2. Batch adsorption studies and isotherm modeling of AC/CS/GO-M membrane

**Fig. 7:**
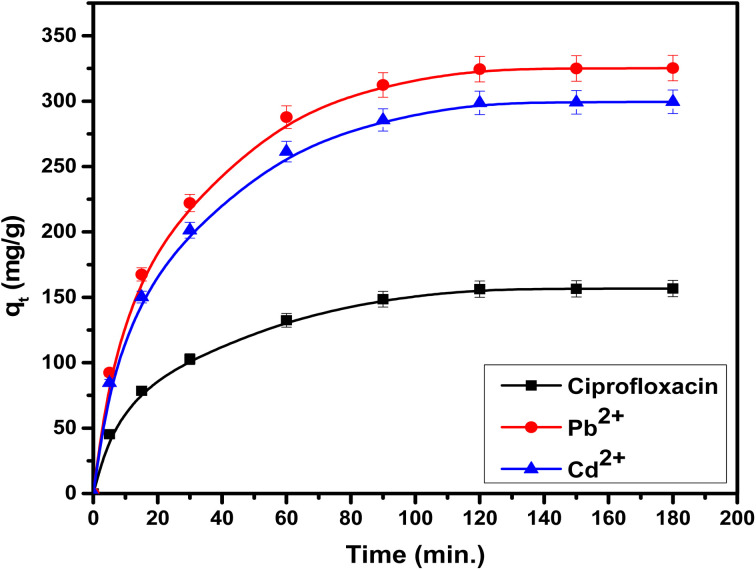
Time-dependent adsorption curves of ciprofloxacin, Pb^2+^, and Cd^2+^ on AC/CS/GO-M membrane.

The adsorption efficacy of the AC/CS/GO-M membrane for the simultaneous removal of ciprofloxacin, Pb^2+^, and Cd^2+^ was evaluated through the use of batch and dynamic filtering tests. The membrane exhibited rapid uptake, with equilibrium reached within 120 minutes for all contaminants according to batch adsorption measurements (Fig. 7). At starting concentrations of 25 mg L^−1^ for ciprofloxacin and 50 mg L^−1^ for the metal ions, the adsorbent showed capacities of 156.2 mg g^−1^ for ciprofloxacin, 324.5 mg g^−1^ for Pb^2+^, and 298.7 mg g^−1^ for Cd^2+^ at pH 6.5 and 25 °C.

**Fig. 8:**
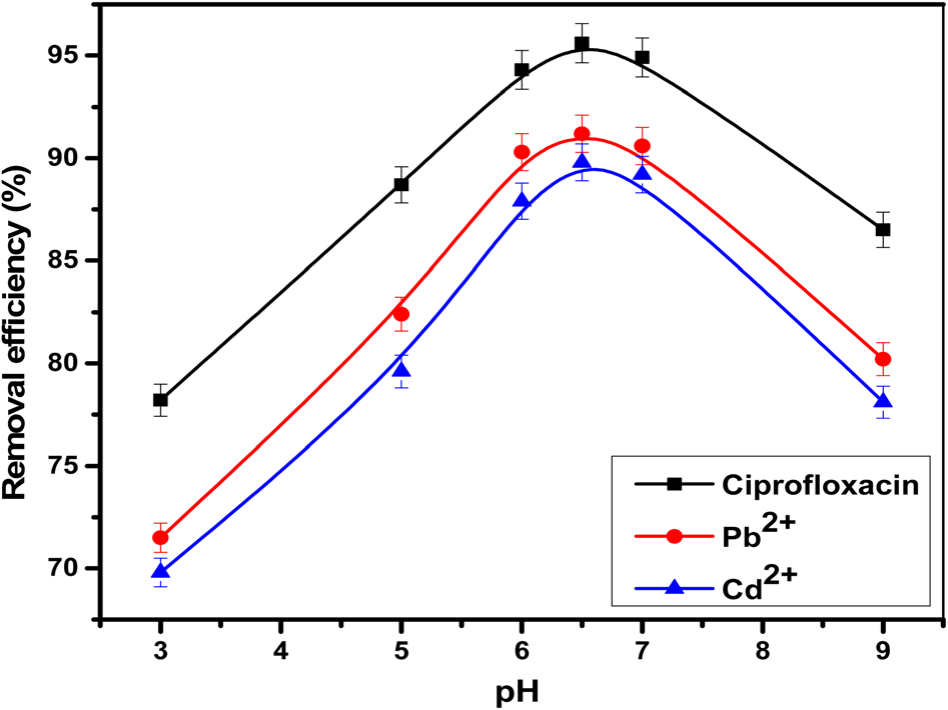
The impact of solution pH on the removal efficiency of ciprofloxacin and heavy metals utilizing the AC/CS/GO-M membrane.

The effects of solution pH were systematically assessed from pH 3 to 9, demonstrating maximum removal efficiency at pH 6–7 for ciprofloxacin and Pb^2+^/Cd^2+^, indicating the most favorable ionization states and electrostatic interactions (Fig. 8).

**Fig. 9:**
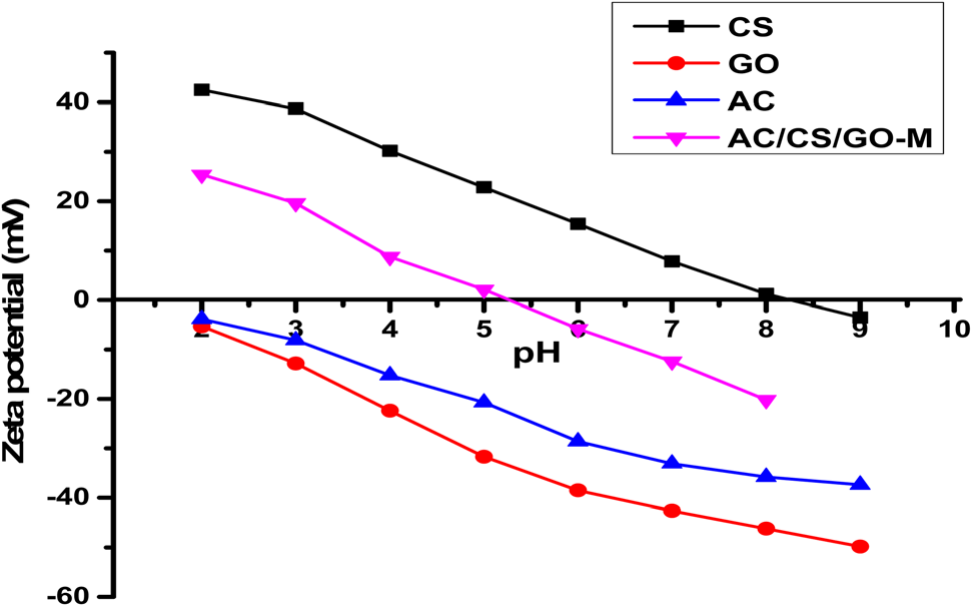
The plots of zeta potential *vs.* pH for graphene oxide (GO), chitosan (CS), activated carbon (AC), and the AC/CS/GO-M membrane.

Fig. 9 shows how the zeta potential values of chitosan (CS), graphene oxide (GO), activated carbon (AC), and the composite membrane (AC/CS/GO-M) change when the pH level changes from 2 to 9. A drop in zeta potential with rising pH is observed in all samples, suggesting that surface functional groups are being deprotonated to a greater extent. At lower pH levels, CS has the highest positive zeta potential because its amino groups are protonated. In contrast, GO and AC both have negative potentials across the pH range, meaning that oxygen-containing groups, such as –COOH and –OH, are present. The AC/CS/GO-M composite presents zeta potential values that lie between those of CS and (AC and GO), showing that the components have been well-integrated and that surface charges have been partially neutralized. Additionally, the isoelectric point (IEP) of the composite shifts to a lower pH in comparison to pure CS, suggesting enhanced surface stability and dispersion in an aqueous environment.

#### 3.2.1. Adsorption kinetics and isotherms

**Fig. 10:**
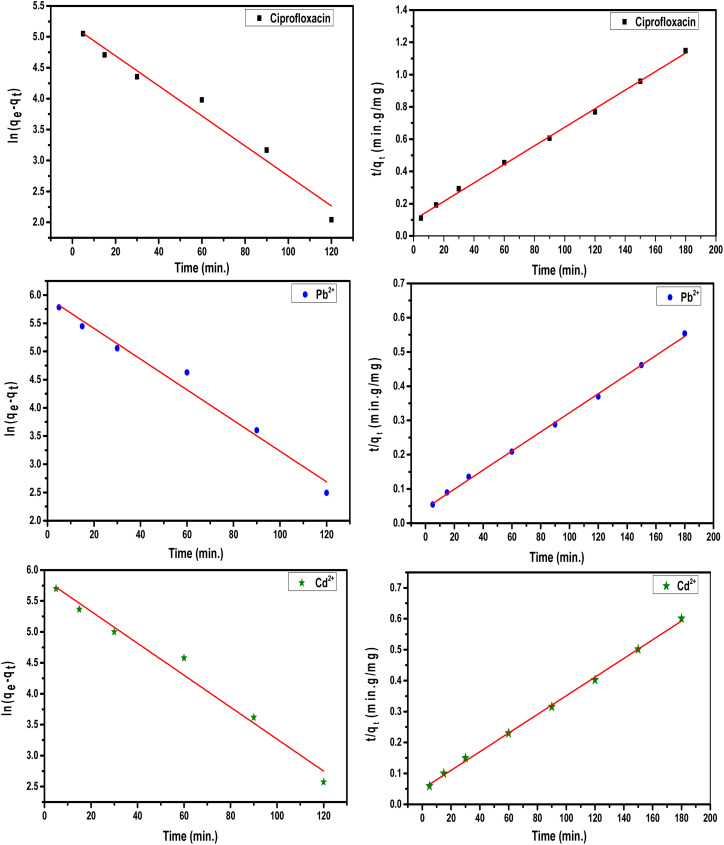
Adsorption kinetic profiles of ciprofloxacin, Pb^2+^, and Cd^2+^ onto the AC/CS/GO-M membrane corresponding to pseudo-first-order and pseudo-second-order models.

Both pseudo-first-order and pseudo-second-order models were used to assess the adsorption kinetics of ciprofloxacin, Pb^2+^, and Cd^2+^ on the AC/CS/GO-M membrane in order to clarify the mechanism and rate-limiting stages of the adsorption process. Time-dependent adsorption studies showed a fast initial uptake within the first 30 minutes, after which the process gradually reached equilibrium around 120 minutes (Fig. 10). In contrast to the pseudo-first-order model, which had *R*^2^ values significantly lower (0.96–0.97) for all three pollutants, the pseudo-second-order model fitted the kinetic data quite well (*R*^2^ values surpassing 0.998). This suggests that the adsorption process is controlled by chemisorption, which is characterized by the sharing or exchange of electrons between the pollutant molecules and the active sites of the adsorbent. The calculated rate constants (*k*_2_) for ciprofloxacin, Pb^2+^, and Cd^2+^ were found to be 0.02, 0.015, and 0.015 g mg^−1^ min^−1^, respectively. These values showed that the adsorption of heavy metals was somewhat faster because of greater coordination interactions with the amino groups in chitosan (Table 3).

**Table 3:** The adsorption kinetic parameters of ciprofloxacin, Pb^2+^, and Cd^2+^ on AC/CS/GO-M according to pseudo-first-order and pseudo-second-order models

Contaminant	*q*_e,exp_ (mg g^−1^)	Pseudo-first-order			Pseudo-second-order		
		*k*_1_ (min^−1^)	*q*_e,cal_ (mg g^−1^)	*R* ^2^	*k*_2_ (g mg^−1^ min^−1^)	*q*_e,cal_ (mg g^−1^)	*R* ^2^
Ciprofloxacin	156.2	0.024	177.57	0.965	0.02	158.73	0.998
Pb^2+^	324.5	0.027	384.89	0.974	0.015	327.87	0.999
Cd^2+^	298.7	0.025	347.56	0.975	0.015	303.03	0.999

**Fig. 11:**
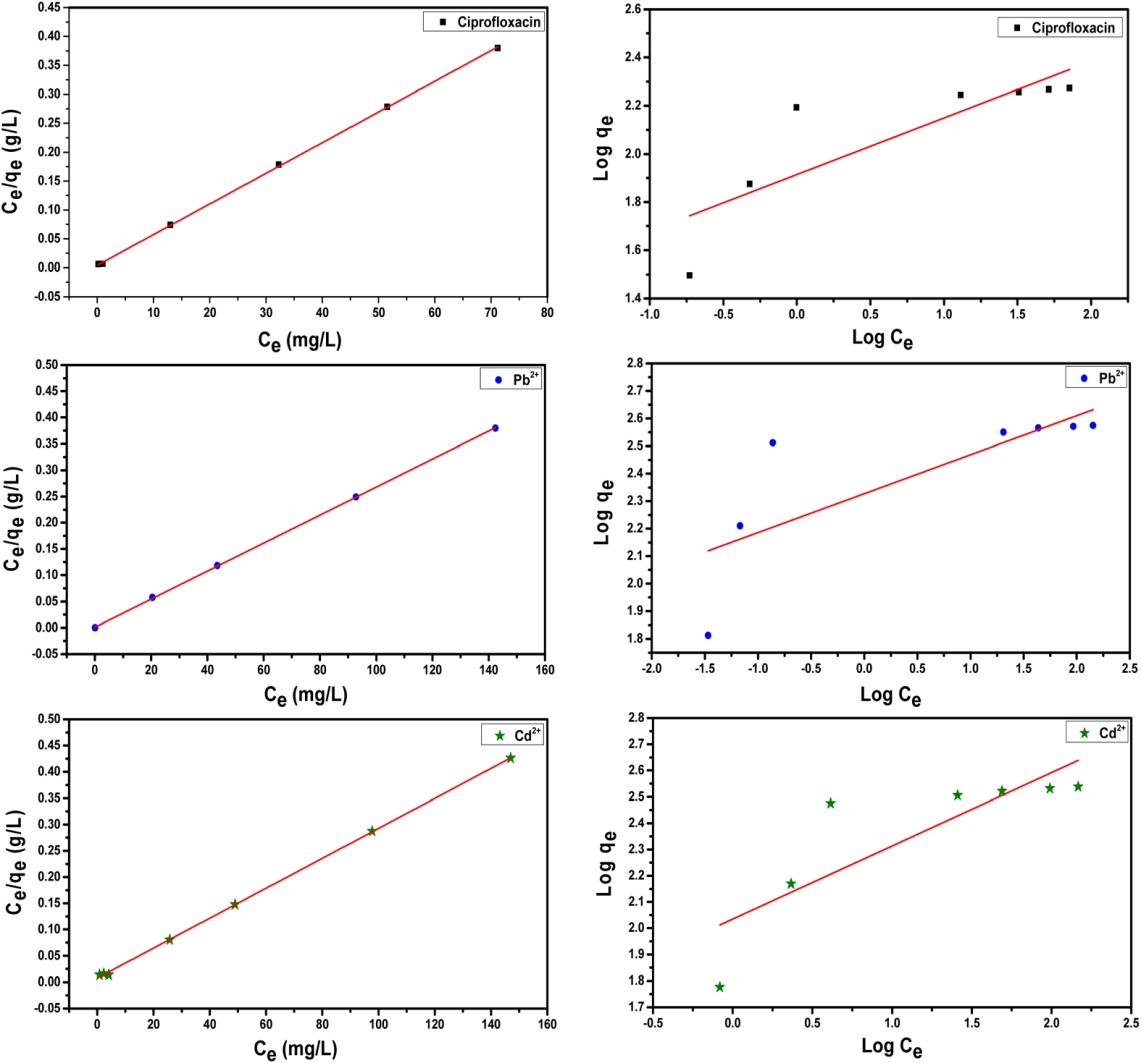
AC/CS/GO-M membrane-based Langmuir and Freundlich adsorption isotherms for ciprofloxacin, Pb^2+^, and Cd^2+^ removal.

To ascertain adsorption capacity and surface affinity, the Freundlich and Langmuir isotherm models were applied to analyze the equilibrium adsorption data. With correlation coefficients of 0.999, the Langmuir model demonstrated better fitting for all three pollutants, suggesting monolayer adsorption on a limited number of uniform sites in the membrane matrix (Fig. 11). In close agreement with the experimentally determined values, the maximal monolayer adsorption capacities (*q*_max_) obtained *via* Langmuir fitting were 161.29 mg g^−1^ for ciprofloxacin, 328.94 mg g^−1^ for Pb^2+^, and 304.87 mg g^−1^ for Cd^2+^. The Langmuir isotherm's separation factor (*R*_L_) demonstrated favorable adsorption within the studied concentration range, with values ranging from 0.65 to 0.92.^37^ Freundlich constants (*n* = 3.59–7.07) further supported the favorable adsorption behaviour and slight surface heterogeneity due to the ternary composition of activated carbon, chitosan, and graphene oxide, as illustrated in Table 4.^38^

**Table 4:** The parameters of AC/CS/GO-M membrane-based Langmuir and Freundlich adsorption isotherms for ciprofloxacin, Pb^2+^, and Cd^2+^ removal

Contaminant	Langmuir			Freundlich		*R* ^2^ (Langmuir/Freundlich)
	*q*_max_ (mg g^−1^)	*a*_L_ (L mg^−1^)	*R*_L_	*n*	*K*_F_ (mg g^−1^ (L mg^−1^)^1/*n*^)	
Ciprofloxacin	161.29	1.47	0.87	4.25	82.09	0.999/0.649
Pb^2+^	328.94	2.65	0.65	7.07	212.40	0.999/0.531
Cd^2+^	304.87	0.43	0.92	3.59	108.37	0.999/0.642

The combined kinetic and isotherm results highlight the high adsorption efficiency of the AC/CS/GO-M membrane, which rapidly captures pollutants through plenty of interactions, comprising π–π stacking, hydrogen bonding, chelation, and electrostatic attraction. The membrane's hierarchical pore structure enables quick mass transfer, and the distributed functional groups offer high-affinity sites for both ionic and organic contaminants. The synergistic benefit of the ternary hybrid design is demonstrated by comparisons with literature data (Table 5), which show the superior adsorption capacity of AC/CS/GO-M over single-component chitosan or activated carbon membranes for ciprofloxacin and heavy metals. These findings support the membrane's dual use as a platform for the simultaneous extraction of hazardous metals and antibiotics from intricate water systems.

**Table 5:** Comparative summary of maximum adsorption capacities (*q*_max_) of AC/CS/GO-M membrane with previously reported chitosan-based, activated carbon-based, and graphene oxide-based adsorbents

Adsorbent	Contaminant	*q*_max_ (mg g^−1^)	References
AC/CS/GO-M (this study)	Ciprofloxacin	156.2	This work
AC/CS/GO-M (this study)	Pb^2+^	324.5	This work
AC/CS/GO-M (this study)	Cd^2+^	298.7	This work
pH-tunable GO/chitosan beads	Ciprofloxacin	119.81	39
PNs composed of alkylated chitosan ionic macromonomers, ionic monomers, and hydrotalcite (HTC)	Ciprofloxacin	84.43	40
Chitosan–graphene oxide (CSN@GO) hybrid beads	Ciprofloxacin	35.36	41
MMT/CS/ZnO hydrogel nanocomposite	Ciprofloxacin	56.49	42
Fe_3_O_4_/graphene oxide/CS nanoparticles	Pb^2+^	63.45	43
MMT/CS/AC composite	Pb^2+^	50	44
Cross-linked alginate–rice husk ash–GO–CS nanoparticles	Pb^2+^	242.5	45
MCS-IHPMB	Pb^2+^	200.11	46
Magnetized activated carbon (MAC)	Pb^2+^	253.2	47
	Cd^2+^	73.3	
CLCTB	Cd^2+^	199.69	48
Magnetic chitosan-mediated GO	Cd^2+^	33.91	49
Chitosan@activated carbon composite	Cd^2+^	84.75	36
Chitosan/nano-hydroxyapatite composite	Cd^2+^	126.65	50

#### 3.2.2. Thermodynamic analysis of adsorption

To comprehend the spontaneity, feasibility, and heat changes related to the adsorption process, the thermodynamic characteristics of ciprofloxacin, Pb^2+^, and Cd^2+^ adsorption onto the AC/CS/GO-M membrane were assessed. The calculated Δ*G*° values were negative for all contaminants and became slightly more negative with increasing temperature, ranging from −23.4 to −27.8 kJ mol^−1^ for ciprofloxacin, −31.2 to −35.5 kJ mol^−1^ for Pb^2+^, and −28.7 to −32.1 kJ mol^−1^ for Cd^2+^, indicating spontaneous adsorption under the studied conditions^51,52^ (Table 6). The positive Δ*H*° values indicate that the adsorption process is endothermic, with values of 42.5 kJ mol^−1^ for ciprofloxacin, 58.2 kJ mol^−1^ for Pb^2+^, and 53.7 kJ mol^−1^ for Cd^2+^. This suggests that higher temperatures are favorable, as they promote increased mobility of ions and enhanced interactions with active sites on the membrane.^51,52^ the higher positive Δ*S*° values (0.22–0.29 kJ mol^−1^ K^−1^) at the solid–liquid boundary indicate higher randomness, which was probably caused by the water molecules' displacement during adsorption and the development of stronger pollutant–membrane interactions.^51,52^

**Table 6:** Ciprofloxacin, Pb^2+^, and Cd^2+^ adsorption thermodynamic parameters

Contaminant	Δ*G*° (kJ mol^−1^)	Δ*H*° (kJ mol^−1^)	Δ*S*° (kJ mol^−1^ K^−1^)	Process nature
Ciprofloxacin	−23.4 to −27.8	42.5	0.22	Spontaneous, endothermic
Pb^2+^	−31.2 to −35.5	58.2	0.29	Spontaneous, endothermic
Cd^2+^	−28.7 to −32.1	53.7	0.27	Spontaneous, endothermic

**Fig. 12:**
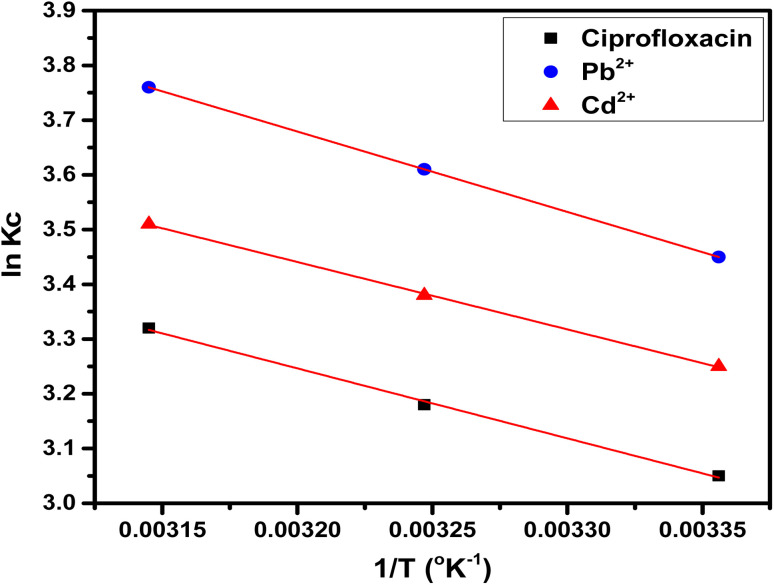
Van't Hoff plots showing ln *K*_c_*vs.* 1/*T* for the adsorption of ciprofloxacin, Pb^2+^, and Cd^2+^ on the AC/CS/GO-M membrane.

The results demonstrate that adsorption of both ciprofloxacin and heavy metals onto AC/CS/GO-M is thermodynamically favorable, spontaneous, and enhanced at higher temperatures. The coexistence of endothermicity with elevated entropy validates that a variety of adsorption processes, comprising chelation, π–π interactions, hydrogen bonding, and electrostatic attraction, occur synergistically on the hierarchical and functionalized membrane surface. High correlation coefficients (*R*^2^ > 0.995) and linear behavior were shown in the Vant Hoff plots of ln *K*_c_*vs.* 1/*T* (Fig. 12), confirming the reliability of the determined thermodynamic parameters. These findings confirm the robustness and applicability of AC/CS/GO-M for advanced water treatment across a range of operational conditions.

### 3.3. Dynamic filtration performance, regeneration, and reusability of AC/CS/GO-M membrane

#### 3.3.1. Dynamic filtration performance and flux stability

**Fig. 13:**
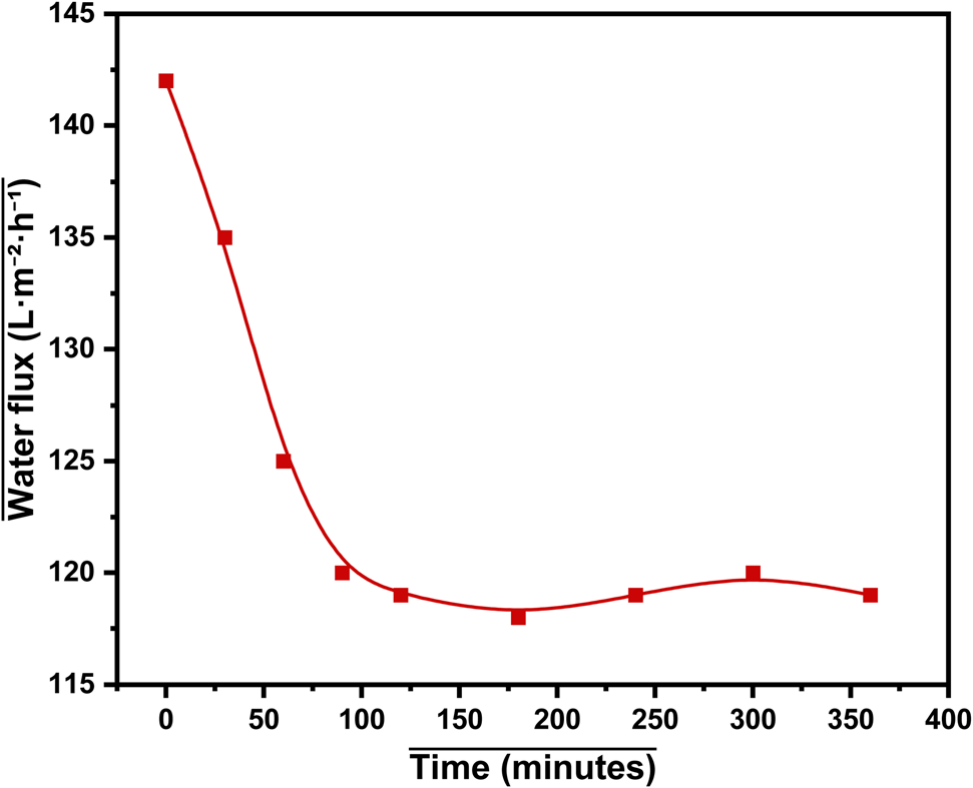
Dynamic filtration performance of the AC/CS/GO-M membrane: water flux as a function of time during continuous 6 hours operation with a feed solution containing ciprofloxacin (25 mg L^−1^), Pb^2+^ (50 mg L^−1^), and Cd^2+^ (50 mg L^−1^) at pH 6.5, 25 °C, and 0.2 MPa transmembrane pressure. Steady-state removal efficiencies achieved after 6 hours were 95.6% ± 1.2% for ciprofloxacin, 91.2% ± 1.5% for Pb^2+^, and 89.8% ± 1.8% for Cd^2+^.

**Fig. 14:**
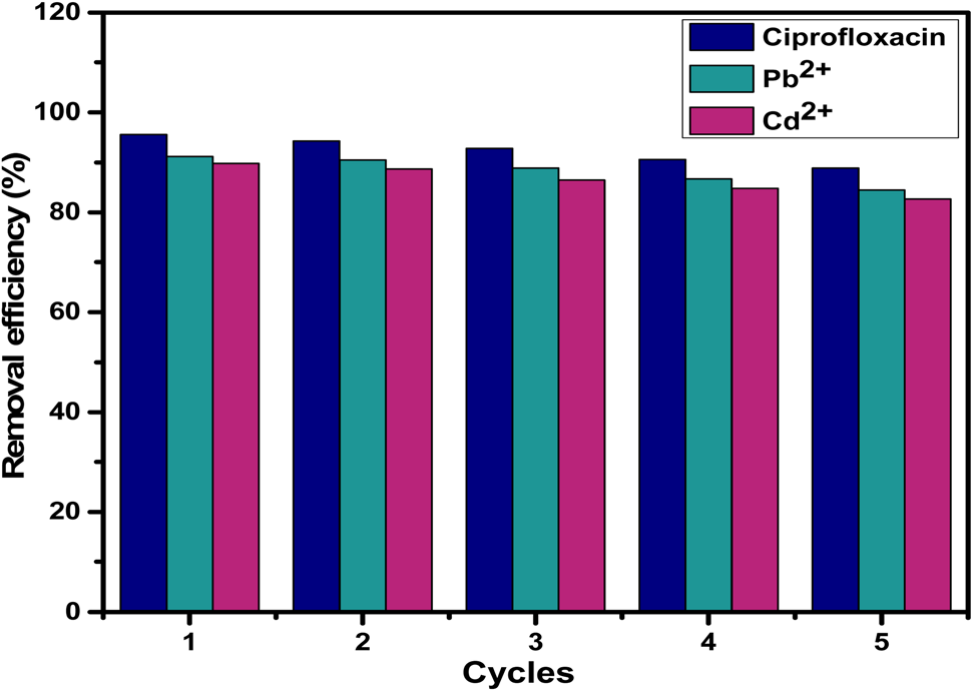
Removal performance of ciprofloxacin, Pb^2+^, and Cd^2+^ over five adsorption–desorption cycles.

Dynamic filtration experiments were conducted using a dead-end filtration cell (Amicon 8200) at a transmembrane pressure of 0.2 MPa and a feed solution containing ciprofloxacin (25 mg L^−1^), Pb^2+^ (50 mg L^−1^), and Cd^2+^ (50 mg L^−1^) at pH 6.5 and 25 °C. Regarding flux stability, the initial pure water flux of the AC/CS/GO-M membrane was 142 L m^−2^ h^−1^. Upon exposure to the pollutant feed solution, the flux decreased progressively over the first 90 minutes to 120 L m^−2^ h^−1^ (a 15.5% decline), after which it stabilized and remained nearly constant at 118–121 L m^−2^ h^−1^ for the subsequent 4.5 hours of operation (Fig. 13). The initial flux decline is attributed to concentration polarization and pore blockage by adsorbed pollutant molecules, particularly ciprofloxacin, which has a larger molecular footprint than the metal ions. Importantly, the flux did not continuously decline, indicating that the membrane surface reached a pseudo-steady state where the rate of pollutant arrival balanced the rate of adsorption/desorption. No irreversible fouling was observed, as evidenced by the complete recovery of initial water flux after a simple 0.1 M NaOH wash (regeneration efficiency > 92% after 5 cycles, Fig. 14). The maintained high flux (≥118 L m^−2^ h^−1^) throughout the 6 hours filtration period, combined with the consistently high rejection rates, demonstrates that the AC/CS/GO-M membrane possesses excellent fouling resistance and operational stability, making it suitable for prolonged wastewater treatment applications.

The steady-state removal efficiencies, determined after 6 hours of continuous operation, were 95.6% ± 1.2% for ciprofloxacin, 91.2% ± 1.5% for Pb^2+^, and 89.8% ± 1.8% for Cd^2+^. These high rejection rates are attributed to a multi-mechanistic barrier effect rather than size exclusion alone, given that the membrane's average pore diameter (12.3 nm) exceeds the molecular dimensions of all three pollutants.

#### 3.3.2. Mechanisms of pollutant removal during dynamic filtration

It is important to emphasize that the removal of ciprofloxacin, Pb^2+^, and Cd^2+^ during dynamic filtration is not governed by adsorption alone but rather by a multi-mechanistic synergistic process involving the following contributions:

(1) Physical size exclusion (sieving): the AC/CS/GO-M membrane possesses a hierarchical porous structure with average pore diameters of ∼12.3 nm (BET analysis, Table 1) and surface pore sizes ranging from 50–150 nm (SEM, Fig. 1). While this pore regime is larger than the molecular dimensions of ciprofloxacin (∼1–2 nm) and hydrated heavy metal ions (∼0.4–0.8 nm), partial steric hindrance and tortuous pathway effects still contribute to retention, particularly for larger aggregates or contaminant clusters.

(2) Electrostatic attraction/repulsion: as demonstrated by the zeta potential measurements (Fig. 9), the AC/CS/GO-M membrane surface carries a pH-dependent charge. At the optimal pH of 6.5, the membrane exhibits a near-neutral to slightly negative surface charge (zeta potential ∼ −15 mV). Under these conditions:

• Ciprofloxacin (p*K*_a1_ ≈ 6.1, p*K*_a2_ ≈ 8.7) exists predominantly as a zwitterion, enabling electrostatic interactions with both positive (protonated –NH_2_) and negative (deprotonated –COOH, –OH) sites on the membrane.

• Pb^2+^ and Cd^2+^ cations are attracted to negatively charged oxygen-containing functional groups (–COO^−^, –O^−^) on GO and AC, as well as to deprotonated hydroxyl groups in chitosan.

(3) Chelation/complexation: the amino (–NH_2_) and hydroxyl (–OH) groups of chitosan serve as excellent chelating ligands for heavy metal ions. During dynamic filtration, as the feed solution permeates through the membrane matrix, Pb^2+^ and Cd^2+^ form stable coordination complexes with these functional groups. This chemisorption process (confirmed by the pseudo-second-order kinetic model, Table 3) is the dominant mechanism for heavy metal removal.

(4) π–π stacking interactions: the sp^2^-hybridized carbon domains of both graphene oxide nanosheets and activated carbon provide abundant aromatic surfaces. Ciprofloxacin, containing a quinoline ring system, interacts strongly with these graphitic regions through π–π stacking. This mechanism is unique to organic pollutants like ciprofloxacin and does not apply to metal ions, explaining the higher removal efficiency observed for ciprofloxacin (>95%) compared to heavy metals (>90%) under identical conditions.

(5) Hydrogen bonding: the oxygen-rich functional groups of GO (C–O–C, –OH, –COOH), the surface oxides of AC, and the –NH_2_/–OH groups of chitosan all participate in hydrogen bonding with:

• The carboxyl and carbonyl groups of ciprofloxacin.

• Hydrated water shells surrounding Pb^2+^ and Cd^2+^ ions.

(6) Synergistic interplay during filtration: unlike static batch adsorption, dynamic filtration continuously brings fresh pollutant molecules to the membrane surface and internal pore walls, reducing boundary layer resistance and enhancing mass transfer. This convective flow accelerates the rate of all the above mechanisms, leading to faster attainment of removal equilibrium compared to batch mode. Furthermore, the applied transmembrane pressure (0.2 MPa) may induce slight membrane compaction, potentially reducing effective pore size and enhancing size exclusion without compromising water flux.

In summary, the high removal efficiencies observed during dynamic filtration (>95% for ciprofloxacin, >90% for Pb^2+^ and Cd^2+^) arise from a concerted action of physical sieving, electrostatic attraction, chelation, π–π stacking, and hydrogen bonding, with adsorption serving as the primary but not exclusive mechanism. This multi-barrier approach is precisely why the AC/CS/GO-M membrane outperforms conventional single-mechanism adsorbents or size-exclusion membranes.

#### 3.3.3. Regeneration and reusability

Following five consecutive filtration–regeneration cycles *via* a simple 0.1 M NaOH wash, the membrane demonstrated excellent stability (Fig. 14). The steady-state efficiency in removing ciprofloxacin, Pb^2+^, and Cd^2+^ declined only slightly from initial values (95.6%, 91.2%, and 89.8%) to (88.9%, 84.5%, and 82.7%), respectively. This minor reduction in performance was attributed to the partial occupation of active sites and slight structural compaction during repeated adsorption–desorption cycles, making the membrane highly suitable for prolonged wastewater treatment applications.

Scanning electron microscopy (SEM) imaging of the membrane after five cycles provided further evidence of its excellent reusability, which revealed that the porous architecture was still largely intact with no discernible aggregation or detachment of graphene oxide nanosheets or activated carbon particles. These findings show that AC/CS/GO-M has outstanding structural and functional stability, allowing for several pollutant removal cycles without noticeably affecting performance. The membrane's strong durability comes from its hierarchical porosity, many functional groups, and strong crosslinking. This makes it a good choice for wastewater treatment applications that require continuous or repeated operation.

Overall, the AC/CS/GO-M membrane exhibited superior adsorption capacity, rapid kinetics, exceptional reusability, and extensive applicability for the concurrent elimination of pharmaceutical contaminants and heavy metals, surpassing previously documented single- or binary-component membranes (Table 5). The membrane is a good choice for practical wastewater treatment applications that target complex pollutant mixtures because of its durability, hydrophilicity, and versatility.

### 3.4. Proposed adsorption mechanisms of ciprofloxacin and heavy metals onto AC/CS/GO-M

**Fig. 15:**
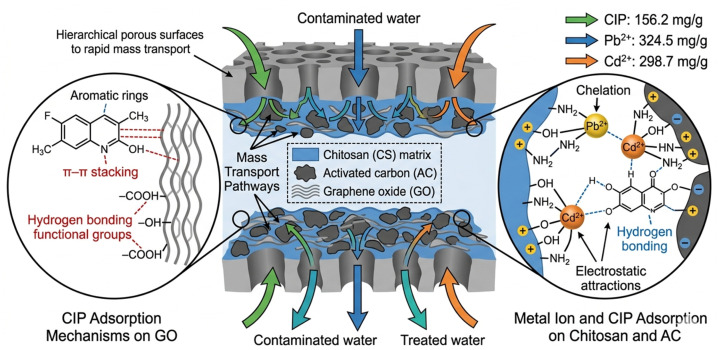
Schematic illustration of the proposed multi-mechanistic adsorption pathways for simultaneous removal of ciprofloxacin (CIP), Pb^2+^, and Cd^2+^ on the AC/CS/GO-M hybrid membrane. The hierarchical porous structure (top/bottom) facilitates rapid mass transport to internal active sites. The central panel shows the membrane composition: chitosan matrix (blue), activated carbon particles (dark gray irregular shapes), and graphene oxide nanosheets (wavy gray layers). Zoomed insets detail specific interactions: (left) π–π stacking between the aromatic rings of ciprofloxacin and the sp^2^ carbon domains of GO, along with hydrogen bonding (dashed lines) between CIP and oxygen-containing functional groups; (right) chelation of Pb^2+^ and Cd^2+^ by amino (–NH_2_) and hydroxyl (–OH) groups of chitosan, with additional hydrogen bonding for CIP. Electrostatic attractions (labeled +/−) are also shown. This synergistic combination of interactions accounts for the high adsorption capacities (CIP: 156.2 mg g^−1^, Pb^2+^: 324.5 mg g^−1^, Cd^2+^: 298.7 mg g^−1^) and rapid kinetics observed.

Based on the comprehensive characterization results (FTIR, XRD, BET, zeta potential, SEM, TEM) and the kinetic, isotherm, and thermodynamic analyses, a multi-mechanistic adsorption pathway is proposed for the simultaneous removal of ciprofloxacin (CIP), Pb^2+^, and Cd^2+^ by the AC/CS/GO-M hybrid membrane. Fig. 15 presents a schematic illustration summarizing the key interactions.

The exceptional adsorption performance of the AC/CS/GO-M membrane arises from the synergistic combination of its three constituent materials, each contributing distinct yet complementary functionalities:

Chitosan (CS): provides abundant amino (–NH_2_) and hydroxyl (–OH) groups that serve as: (i) chelation sites for heavy metal ions (Pb^2+^, Cd^2+^) *via* coordinate covalent bonds; (ii) hydrogen bond donors/acceptors for ciprofloxacin's carbonyl and carboxyl groups; and (iii) electrostatic binding sites when protonated (–NH_3_^+^) at lower pH.

Graphene oxide (GO): contributes large sp^2^-hybridized aromatic domains for π–π stacking with the quinoline ring system of ciprofloxacin, as well as oxygen-containing functional groups (–COOH, –OH, C–O–C) that participate in hydrogen bonding and electrostatic interactions. The two-dimensional nanosheet morphology also provides a high surface area and acts as a barrier against polymer chain mobility, enhancing mechanical stability.

Activated carbon (AC): provides a highly porous backbone (BET surface area contribution) with extensive micro- and mesopores that trap pollutants *via* physical entrapment and van der Waals forces. Its graphitic domains further contribute to π–π stacking, while surface oxygen groups (introduced during activation) enable hydrogen bonding.

The hierarchical porous structure (pore diameters ranging from ∼2 nm to ∼200 nm) facilitates rapid mass transfer of pollutants to internal active sites, reducing diffusion limitations and enabling fast adsorption kinetics (equilibrium within 120 minutes).

As illustrated in Fig. 15, the following specific interactions occur simultaneously:

• For ciprofloxacin: π–π stacking between its aromatic quinoline and piperazinyl rings and the graphitic domains of GO and AC; hydrogen bonding between its –COOH, <svg xmlns="http://www.w3.org/2000/svg" version="1.0" width="10.400000pt" height="16.000000pt" viewBox="0 0 10.400000 16.000000" preserveAspectRatio="xMidYMid meet"><metadata> Created by potrace 1.16, written by Peter Selinger 2001-2019 </metadata><g transform="translate(1.000000,15.000000) scale(0.011667,-0.011667)" fill="currentColor" stroke="none"><path d="M80 1160 l0 -40 40 0 40 0 0 -40 0 -40 40 0 40 0 0 -40 0 -40 40 0 40 0 0 -40 0 -40 40 0 40 0 0 -40 0 -40 40 0 40 0 0 -40 0 -40 40 0 40 0 0 -40 0 -40 40 0 40 0 0 80 0 80 -40 0 -40 0 0 40 0 40 -40 0 -40 0 0 40 0 40 -40 0 -40 0 0 40 0 40 -40 0 -40 0 0 40 0 40 -40 0 -40 0 0 40 0 40 -80 0 -80 0 0 -40z M560 520 l0 -40 -40 0 -40 0 0 -40 0 -40 -40 0 -40 0 0 -40 0 -40 -40 0 -40 0 0 -40 0 -40 -40 0 -40 0 0 -40 0 -40 -40 0 -40 0 0 -40 0 -40 -40 0 -40 0 0 -40 0 -40 80 0 80 0 0 40 0 40 40 0 40 0 0 40 0 40 40 0 40 0 0 40 0 40 40 0 40 0 0 40 0 40 40 0 40 0 0 40 0 40 40 0 40 0 0 80 0 80 -40 0 -40 0 0 -40z"/></g></svg> C=O, and –NH_2_ groups and the –OH/–NH_2_ groups of chitosan and GO; electrostatic attraction between its zwitterionic form (at pH 6.5) and charged membrane surface sites.

• For Pb^2+^ and Cd^2+^: chelation/complexation with –NH_2_ and –OH groups of chitosan (primary mechanism); electrostatic attraction to negatively charged –COO^−^ and –O^−^ groups on GO and AC; ion exchange with protons on surface functional groups.

The coexistence of ciprofloxacin and heavy metals does not significantly compete for binding sites because they target different functional groups: metals preferentially bind to –NH_2_ (*via* lone pair donation), while ciprofloxacin favors π–π stacking and hydrogen bonding. This site selectivity explains the simultaneous high removal efficiencies observed.

## 4. Conclusion

In this research, a novel activated carbon–chitosan–graphene oxide (AC/CS/GO-M) composite membrane was successfully synthesized through solution casting and crosslinking, demonstrating an increased surface area, numerous functional groups, and hierarchical porosity. Extensive characterization using SEM, FTIR, XRD, BET, TGA, and contact angle analysis verified that graphene oxide and activated carbon were uniformly distributed throughout the chitosan matrix, offering improved adsorption sites, hydrophilicity, and structural integrity. Both batch and dynamic adsorption tests showed that, under ideal conditions (pH 6.5, 25 °C, and 120 min contact time), AC/CS/GO-M had high removal efficiencies for ciprofloxacin (>95%) and heavy metals (Pb^2+^ > 90%, Cd^2+^ > 90%). The respective adsorption capacities for ciprofloxacin, Pb^2+^, and Cd^2+^ were 156.2 mg g^−1^, 324.5 mg g^−1^, and 298.7 mg g^−1^, respectively. A schematic illustration summarizing the multi-mechanistic adsorption pathways—including π–π stacking, hydrogen bonding, electrostatic attraction, and chelation—is presented in Fig. 15, providing a clear visual framework for understanding the synergistic functionality of the ternary hybrid membrane.

The findings from kinetic and isotherm studies demonstrated that the adsorption process aligns with the Langmuir isotherm and follows a pseudo-second-order kinetic model, suggesting the occurrence of chemisorption and monolayer adsorption on homogeneous active sites. Thermodynamic investigations validated that the process was characterized by being entropy-driven, spontaneous, and endothermic, while regeneration and reusability tests showed that the membrane retained outstanding removal performance and structural integrity even after undergoing five consecutive adsorption–desorption cycles. Thanks to the synergistic combination of activated carbon, chitosan, and graphene oxide, the membrane has multifunctional adsorption capabilities, mechanical strength, and excellent water permeability, making it better than many previously reported single- or dual-component membranes.

In summary, the AC/CS/GO-M membrane demonstrates significant durability and efficiency in the simultaneous removal of pharmaceutical contaminants and harmful heavy metals. Although the present study establishes a foundational proof-of-concept using laboratory-prepared water, the membrane's performance and versatility mark it as a promising candidate for advanced wastewater applications. Subsequent work will focus on evaluating the membrane against complex matrix effects, additional micropollutants, and long-term field testing to bridge the gap toward industrial-scale implementation.

## Conflicts of interest

The authors declare that they have no known competing financial interests or personal relationships that could have appeared to influence the work reported in this paper.

## Data Availability

Data will be available upon request. Supplementary information (SI) is available. See DOI: https://doi.org/10.1039/d5ra08891g.
